# New Drug Candidate Targeting the 4A1 Orphan Nuclear Receptor for Medullary Thyroid Cancer Therapy

**DOI:** 10.3390/molecules23030565

**Published:** 2018-03-02

**Authors:** Lei Zhang, Wen Liu, Qun Wang, Qinpei Li, Huijuan Wang, Jun Wang, Tieshan Teng, Mingliang Chen, Ailing Ji, Yanzhang Li

**Affiliations:** 1 Henan University Joint National Laboratory for Antibody Drug Engineering, Kaifeng 475004, China; zhlei@henu.edu.cn (L.Z.); juanjuan5891@163.com (H.W.); xiaoshan1220@163.com (T.T.); chml163@163.com (M.C.); 2 Henan University Medical bioinformatics institute, Kaifeng 475004, China; 3 Henan University School of Basic Medical Sciences, Kaifeng 475004, China; 18236506709@163.com (W.L.); wangqun011@163.com (Q.W.); paopaohaitunyin@163.com (Q.L.); 10190009@vip.henu.edu.cn (J.W.)

**Keywords:** orphan nuclear receptor 4A1 (NR4A1), thyroid cancer, 2-imino-6-methoxy-2*H*-chromene-3-carbothioamide (IMCA)

## Abstract

Medullary thyroid cancer (MTC) is a relatively rare thyroid cancer responsible for a substantial fraction of thyroid cancer mortality. More effective therapeutic drugs with low toxicity for MTC are urgently needed. Orphan nuclear receptor 4A1 (NR4A1) plays a pivotal role in regulating the proliferation and apoptosis of a variety of tumor cells. Based on the NR4A1 protein structure, 2-imino-6-methoxy-2H-chromene-3-carbothioamide (IMCA) was identified from the Specs compounds database using the protein structure-guided virtual screening approach. Computationally-based molecular modeling studies suggested that IMCA has a high affinity for the ligand binding pocket of NR4A1. MTT [3-(4,5-dimethyl-2-thiazolyl)-2,5-diphenyl-2-H-tetrazolium bromide] and apoptosis assays demonstrated that IMCA resulted in significant thyroid cancer cell death. Immunofluorescence assays showed that IMCA induced NR4A1 translocation from the nucleus to the cytoplasm in thyroid cancer cell lines, which may be involved in the cell apoptotic process. In this study, the quantitative polymerase chain reaction results showed that the IMCA-induced upregulation of sestrin1 and sestrin2 was dose-dependent in thyroid cancer cell lines. Western blot showed that IMCA increased phosphorylation of adenosine 5′-monophosphate-activated protein kinase (AMPK) and decreased phosphorylation of ribosomal protein S6 kinase (p70S6K), which is the key enzyme in the mammalian target of rapamycin (mTOR) pathway. The experimental results suggest that IMCA is a drug candidate for MTC therapy and may work by increasing the nuclear export of NR4A1 to the cytoplasm and the tumor protein 53 (p53)-sestrins-AMPK-mTOR signaling pathway.

## 1. Introduction

Medullary thyroid cancer (MTC) is a relatively uncommon cancer of the thyroid C cells, with about 1400 new cases per year in the U.S., yet accounts for a substantial proportion of thyroid cancer mortality [1]. Many studies have focused on MTC treatment, such as, proto-oncogene tyrosine-protein kinase receptor Ret (RET) inhibitors, Ras-like protein 1 (RAS) inhibitors, immunotherapy, and peptide receptor radionuclides. Two RET inhibitors, vandetanib and cabozantinib, are U.S. Food and Drug Administration (FDA)-approved for the treatment of advanced MTC [2,3]. MTC is resistant to cytotoxic chemotherapy. Developing more effective drugs with higher bioactivity and fewer toxic side effects is urgently required for MTC treatment. The orphan nuclear receptor 4A1 (NR4A1) plays a pivotal role in regulating the proliferation and apoptosis in a variety of tumor cells [4]. NR4A1 may have potential for the development of new drugs for MTC treatment.

NR4A1 is a member of NR4A orphan nuclear receptor family that includes NR4A1, NR4A2, and NR4A3. NR4A1, which includes mouse homologue neuron-derived clone 77 (Nur77), human homologue testicular receptor 3 (TR3), and rat homologue nerve growth factor-induced gene B (NGFI-B), is involved in multiple physiological and pathological processes in metabolism, inflammation, vascular function, and steroidogenesis [5,6,7]. NR4A1 is expressed in most tumor tissues and cells. Functional studies of receptor knockdown caused by RNA interference suggest that NR4A1 knockdown reduces cellular proliferation and angiogenesis and induces intrinsic and extrinsic apoptotic pathways [8]. For instance, inhibition of NR4A1 results in decreased proliferation and increased apoptosis of pancreatic cancer cells [6]. The dual role of NR4A1 in the process of proliferation and apoptosis varies depending on the change in its intracellular location [8]. Compared with non-tumor tissues, NR4A1 is strongly expressed in the nucleus of tumor tissues, whereas inhibition of the expression of NR4A1 may decrease cell proliferation and increase cell apoptosis [9]. Translocation of NR4A1 from the nucleus to the mitochondria is a major event in NR4A1-mediated apoptosis [10]. Pro-apoptotic agents related to NR4A1, such as phorbol esters and adamantly-derived retinoids mainly induce NR4A1 expression and its translocation from the nucleus to mitochondria. NR4A1 binds with B-cell lymphoma-2 (BCL-2) to form a pro-apoptotic complex and triggers the release of cytochrome c and apoptosis [11,12]. NR4A1 is also involved in cell proliferation regulated by the mTOR signaling pathway, which is a main regulator of cell growth and metabolism [13]. Deregulation of the mTOR pathway has been closely linked to tumorigenesis [14]. In lung cancer cells, NR4A1 binds to and deactivates p53, resulting in the activation of mTOR due to decreased expression of p53-regulated sestrin1 and sestrin2 and inactivation of AMPKa [15,16,17]. In summary, for cancer cells, NR4A1 acts as a survival factor in the nucleus, but transforms into a killer after migrating to mitochondria. In the mitochondria, NR4A1 can interact with BCL-2, an anti-apoptotic protein, and lead to the conversion of BCL-2 from a protector to a killer that triggers the release of cytochrome c and apoptosis.

No appropriate ligand-binding cavity exists in NR4A1 to bind small molecular ligands and regulate physiological function. A review of previous studies shows that the regulation activity of NR4A receptors is ligand independent, and NR4A1 function is dependent on receptor expression and posttranslational modification [18]. However, a number of small molecule NR4A1 inhibitors of NR4A1 have been found, such as cytosporone B and related analogs [19,20], (ethyl 2-(2,3,4-trimethoxy-6-(1-octanoyl) phenyl) acetate and 1-(3,4,5-trihydroxyphenyl) no-nan-1-one), 1,1-bis(3′-indolyl)-1-(*p*-substituted phenyl) methane (C-DIM) analogs [21], 1,1-bis-(3′-indolyl)-1-(*p*-hydroxyphenyl)methane (DIM-C-pPhOH) [22,23], and ethyl 2-[2,3,4-trimethoxy-6-(1-octanoyl)phenyl]acetate (TMPA) [24]. Zhan et al first reported the crystal structure of NR4A1, and successfully screened NR4A1 small molecule ligand TMPA, which plays an important role in hepatic gluconeogenesis process mediated by NR4A1, using computer virtual screening technology [24].

In this study, IMCA was obtained from the Specs compounds database using a protein structure-guided virtual screening approach based on the NR4A1 protein structure. We focused on the effect of IMCA on thyroid cancer cell viability, apoptosis, nuclear export of NR4A1, and cell proliferation regulated by the mTOR pathway. The results demonstrate that IMCA is an anticancer drug candidate for thyroid carcinoma chemotherapy and may work as a specific antagonist of NR4A1 through the nuclear export of NR4A1 and the p53-sestrins-AMPK-mTOR signaling pathway.

## 2. Results

### 2.1. Computation-Based IMCA Binding and Interaction with NR4A1

**Figure 1:**
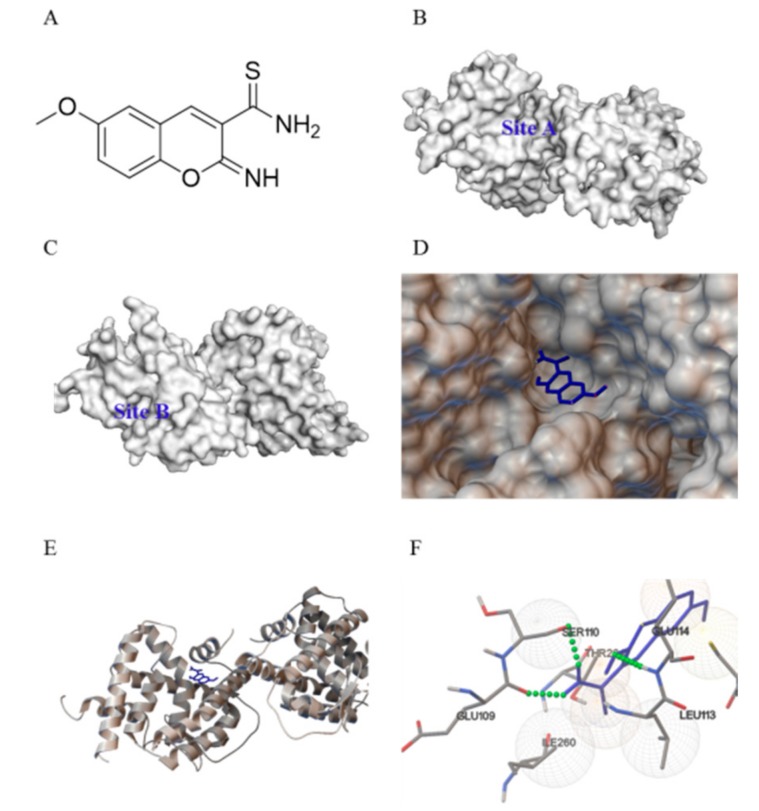
Predicted interaction between the orphan nuclear receptor 4A1 (NR4A1) and 2-imino-6-methoxy-2H-chromene-3-carbothioamide (IMCA). (**A**) Molecular structure of IMCA; (**B**) the ligand binding Site A on the face of the NR4A1 ligand binding domain (LBD); and (**C**) the ligand binding Site B on the face of the NR4A1 LBD; (**D**,**E**) The predicted binding of IMCA (blue) within the ligand binding Site A of the NR4A1 LBD. In this, (**D**) shows the surface of NR4A1; and (**E**) shows the secondary structure of NR4A1; (**F**) Specific non-bonded interactions between IMCA (blue) and the NR4A1 residues (gray). Green dashed lines indicate predicted hydrogen bonds between IMCA and Glu109, Ser110, and Glu114 of the NR4A1 LBD.

The analysis of the complex crystal structure of 3V3Q ligand binding established that two separate and distinct ligand binding sites exist on different faces of the NR4A1 ligand binding domain (LBD) [24]. Site A (Figure 1B), which was composed of helices 4, 11, and 12, is a relatively shallow cavity. The amino acid residues of Site A include Glu109, Ser110, Ala111, Phe112, Leu113, Glu114, Arg184, Leu231, Arg232, Cys235, Thr236, Leu239, Ile260, Thr264, and Phe261. Site B (Figure 1C), which was composed of helices 1, 5, 7, and 8, was also a relatively shallow cavity. The amino acid residues of Site B include His41, Leu42, Pro46, Arg119, Tyr122, Arg123, Lys125, Leu162, His163, Leu165, Leu166, Val167, and Val169. IMCA (Figure 1A) was obtained by screening the binding with Site A, so the docking between IMCA and Site A was calculated. The result indicated that the NR4A1 ligand binding pocket is capable of accommodating IMCA (Figure 1D,E), and the modeling studies predicted that binding occurred in an orientation similar to that reported for the compound TMPA. This binding involves three hydrogen bonds with Glu109, Ser110, and Glu114 (Figure 1F).

### 2.2. IMCA Inhibits Cell Proliferation and Induces Apoptosis in Thyroid Carcinoma Cells

**Figure 2:**
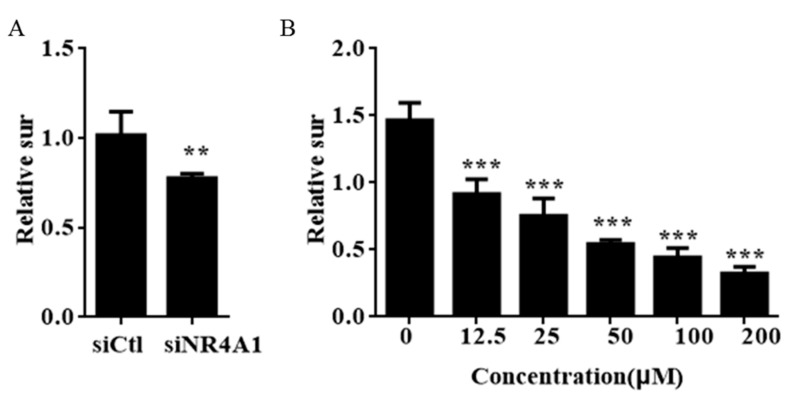
siNR4A1 and IMCA inhibit cell growth in TT cells. (**A**) TT cells were transfected with siCtl or siNR4A1 for 48h and the number of cells were then counted; (**B**) TT cells were treated with 200 μM, 100 μM, 50 μM, 25 μM, and 12.5 μM IMCA for 48 h, and the number of cells were analyzed using the MTT method. Results are means ± standard deviation for at least three separate determinations for each group. The number of cells were lower in the group treated with siRNA or IMCA. ** *p* < 0.01; *** *p* < 0.001.

Through a preliminary virtual screening experiment, we inferred IMCA interacts with NR4A1 and regulates the activation of NR4A1. The effect of NR4A1 on the growth and survival was investigated using the short-interfering RNA (siRNA) (Figure S1) method in thyroid cancer cell lines. The cell survival was significantly inhibited in thyroid cancer cell with low expression of NR4A1 (Figure 2A). Interestingly, MTT experiments confirmed that IMCA significantly induces cell death of TT cells after treatment for 48 h (Figure 2B) and the half maximal inhibitory concentration (IC_50_) value was 13.18 μM.

**Figure 3:**
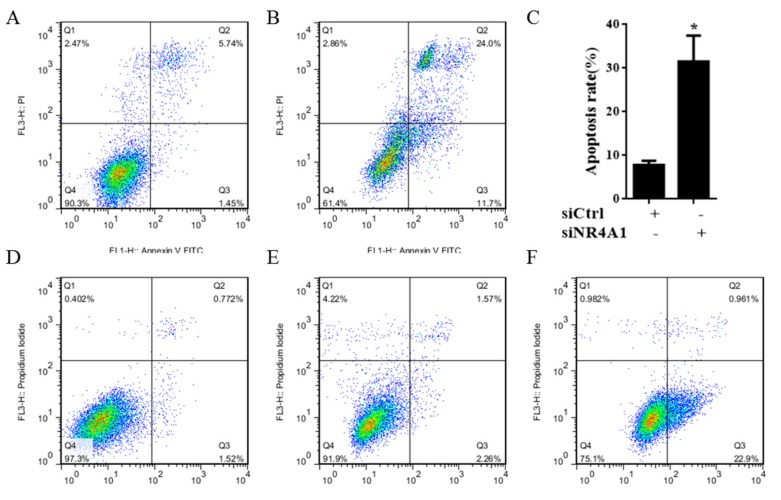

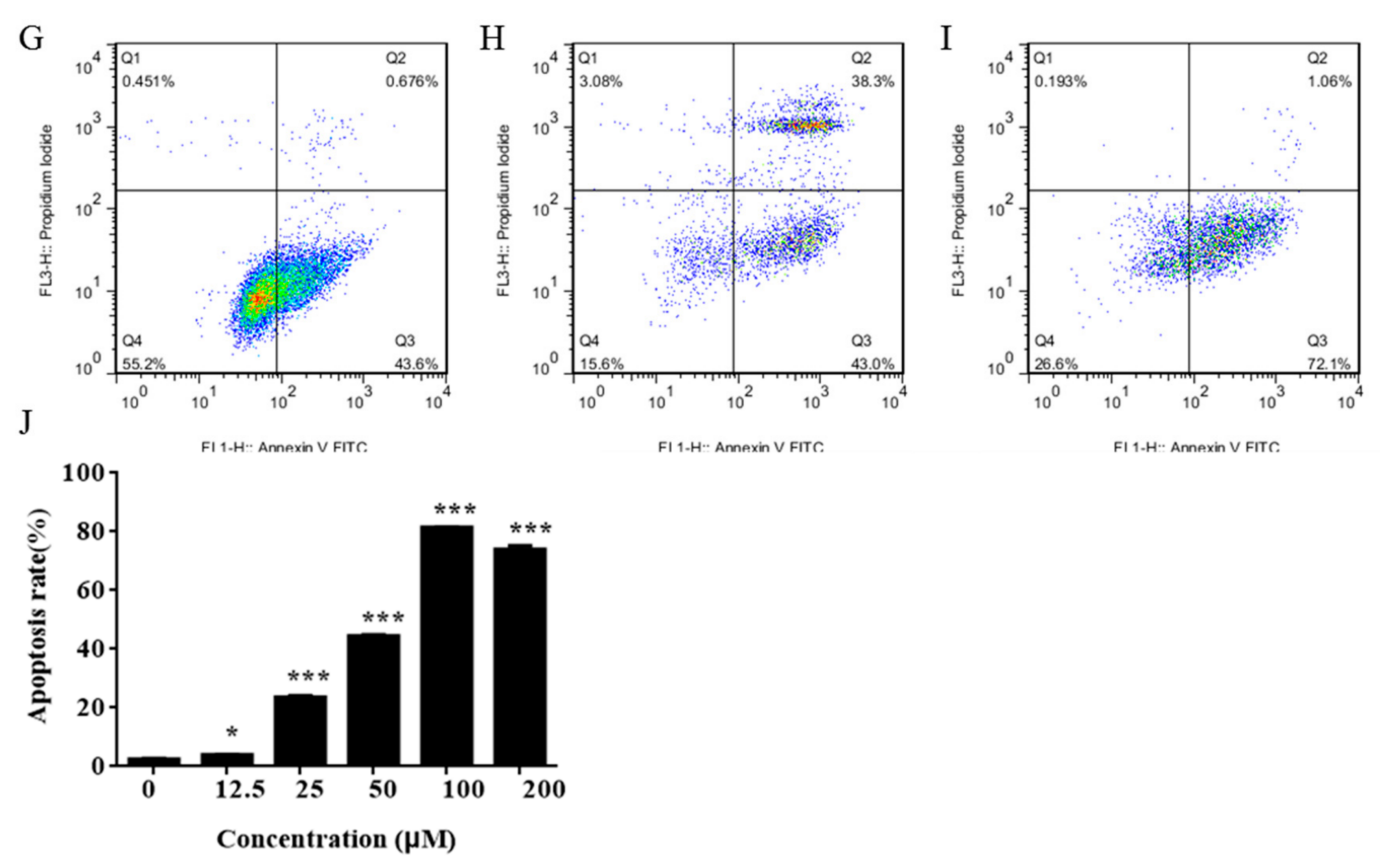
siNR4A1 and IMCA induce apoptosis in TT cells after 48 h. (**A**) Apoptosis induced with siCtrl is detected using flow cytometry in TT cells; (**B**) Apoptosis induced with siNR4A1 is detected using flow cytometry in TT cells; (**C**) Apoptosis induced with siNR4A1 was statistical analyzed in TT cells; (**D**) Apoptosis was detected using flow cytometry in TT cells; (**E**) Apoptosis induced with 12.5 μM IMCA was detected using flow cytometry in TT cells; (**F**) Apoptosis induced with 25 μM IMCA was detected using flow cytometry in TT cells; (**G**) Apoptosis induced with 50 μM IMCA was detected using flow cytometry in TT cells; (**H**) Apoptosis induced with 100 μM IMCA was detected using flow cytometry in TT cells; (**I**) Apoptosis induced with 200 μM IMCA was detected using flow cytometry in TT cells; (**J**) Apoptosis induced with different concentrations of IMCA was analyzed in TT cells. * *p* < 0.05; *** *p* < 0.001.

NR4A1 also regulates the pro-survival genes and pathways in many cancer cells, including thyroid carcinoma cells [4]. Figure 3A–C shows that transfection of TT thyroid carcinoma cells with siNR4A1 induced apoptosis. To confirm that cell death was induced by IMCA through the apoptosis pathway, the effect of IMCA on apoptosis was detected using Annexin V and propidium iodide (PI) staining in TT cells. IMCA significantly exacerbated the apoptosis rate, which was expressed by the mean value of two repetitions of the apoptosis determination (3.36% of the control group, 76.19% in the group treated at an IMCA concentration of 100 μM, 73.10% in the group treated with IMCA at a concentration of 50 μM, 59.38% in the group treated with IMCA at a concentration of 25μM, 33.07% in the group treated with IMCA at a concentration of 12.5 μM, and 6.63% in the group treated with IMCA at a concentration of 6.25 μM) (Figure 3B,E–J). Western blot results showed that the decrease in IMCA concentration was accompanied by elevated expression of the anti-apoptotic BCL-2 and a reduced expression of the apoptotic BCL-2-like protein 4 (BAX). 

**Figure 4:**
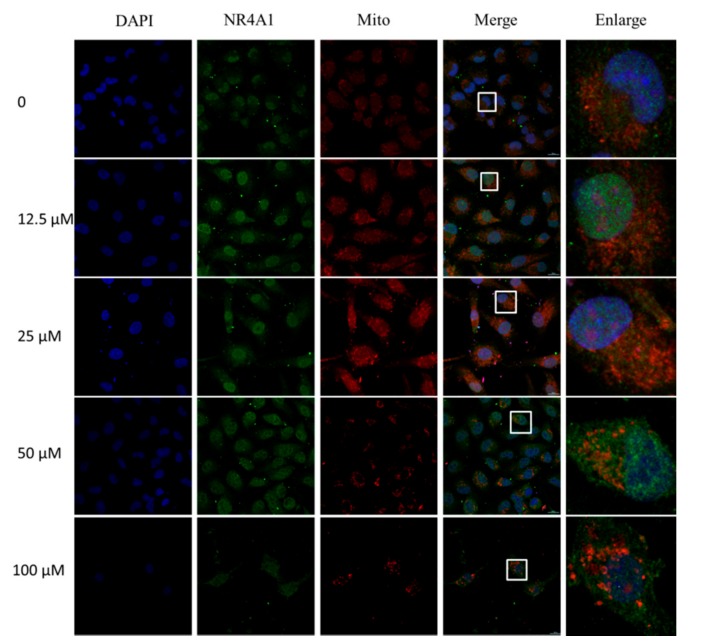
Immunofluorescence and mitochondrial staining assay for the localization of NR4A1 into mitochondria induced by IMCA in TT cells. The TT cells treated with different concentrations of IMCA for 48 h, were stained with 200 nM Mito Tracker^TM^ Red CMXRos-Special Pcakaging, fixed with neutral formalin, and incubated with NR4A1 antibody. Secondary antibody conjugated Alexa Fluor 488 and 4′,6-diamidino-2-phenylindole (DAPI) were added. Fluorescence microscopy showed that the nucleus dyed with DAPI displayed blue fluorescence, NR4A1 immunofluorescence was displayed as green, and mitochondria were displayed as red. The merged images showed that NR4A1 is induced by IMCA to locate to the mitochondria. The graphs in the last column are the magnified images of the white line frame in the fourth column.

Some of the earliest studies of NR4A1 in cancer cells demonstrated the novel pathway in which the caged retinoid compound CD437, several analogs, and diverse apoptosis-inducing agents caused apoptosis in cancer cell lines by inducing nuclear export of NR4A1 [25,26,27]. The nuclear export pathway was linked to the formation of a proapoptotic mitochondrial NR4A1-BCL-2 complex, which was also observed using peptide mimics and paclitaxel which simulates NR4A1 interactions with BCL-2 [11,27,28]. To confirm that IMCA induced cell apoptosis is related to the nuclear export of NR4A1, we detected the nucleoplasm localization using immunofluorescence and the mitochondrial localization using Mito Tracker Red staining. The results showed that IMCA significantly exacerbated the nuclear export and mitochondrial localization of NR4A1 in a dose-dependent manner (Figure 4). 

### 2.3. IMCA Inhibits mTOR Signaling

**Figure 5:**
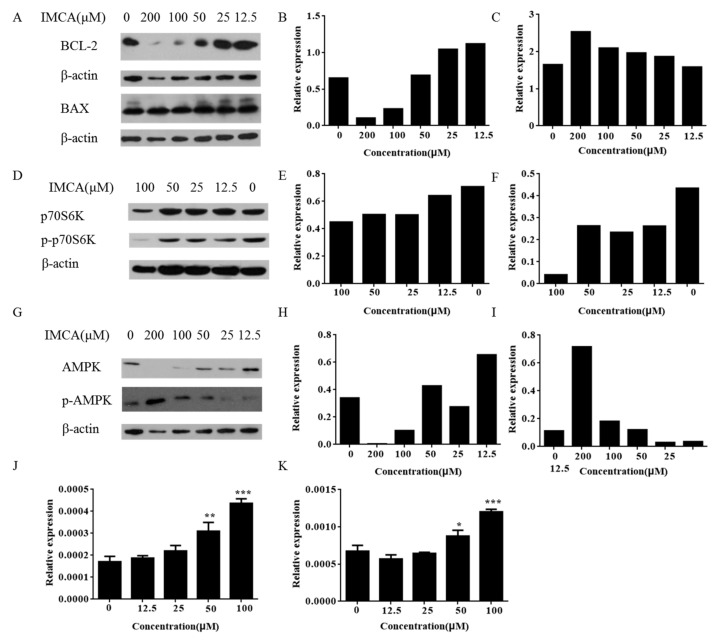
IMCA induced apoptosis and inhibited the mTOR pathway. (**A**) TT cells were treated with IMCA for 48 h and whole cell lysates were analyzed by Western blotting for apoptosis gene products; (**B**) The relative expression of BCL-2 is represented by the gray value ratio of the BCL-2 to its corresponding actin; (**C**) The relative expression of BAX is represented by the gray value ratio of the BAX to its corresponding actin; (**D**) TT cells were treated with IMCA for 48 h and whole cell lysates were analyzed by Western blotting for phosphorylation of p70S6K protein, which is a downstream protein of mTOR; (**E**) The relative expression of p70S6K is represented by the gray value ratio of the p70S6K to its corresponding actin; (**F**) The relative expression of p-p70S6K is represented by the gray value ratio of the p-p70S6K to its corresponding actin; (**G**) TT cells were treated with IMCA for 48 h and whole cell lysates were analyzed by Western blotting for phosphorylation of AMPK; (**H**) The relative expression of AMPK is represented by the gray value ratio of the AMPK to its corresponding actin; (**I**) The relative expression of p-AMPK is represented by the gray value ratio of the p-AMPK to its corresponding actin. TT cells were treated with IMCA for 48 h and whole cell lysates were analyzed by real-time fluorescent quantitative polymerase chain reaction (qPCR) for (**J**) sestrin1 and (**K**) sestrin2. * *p* < 0.05; ** *p* < 0.01; *** *p* < 0.001.

The mTOR signaling pathway is the main regulator of cell growth and metabolism. To explore medullary thyroid cancer cell death induced by IMCA implicated in the mTOR signaling, we examined the key proteins expressed in the mTOR pathway. Western blot analysis showed that IMCA decreased the phosphorylation of p70S6K, a mTOR downstream gene (Figure 5D–F). These results are consistent with previous studies of breast cancer cell lines, in which 1,1-bis(3′-indolyl)-1-(*p*-cyanophenyl)methane (DIM-CpPhCN), a NR4A1 antagonist, decreased the phosphorylation of mTOR and mTOR downstream genes [23]. Furthermore, we observed that the phosphorylation of AMPK was significantly upregulated in TT cells treated with IMCA (Figure 5G–I).Quantiative polymerase chain reaction (qPCR) showed that the expression of sestrin1 and sestrin2 was significantly upregulated in a dose-dependent manner, in TT cells treated with IMCA (Figure 5J,K). These results are consistent with previous studies in lung cancer cell lines where the inactivation of NR4A1 activated p53-dependent activation of *sesntrin2* which activated AMPKa [19]. Thus, IMCA inhibits nuclear NR4A1-mediated mTOR pathways and represent a new class of mechanism-based drugs for thyroid carcinoma chemotherapy.

## 3. Discussion

The orphan nuclear receptor NR4A1 is an immediate early gene whose expression is induced by multiple stressors, playing an important role in maintaining cellular homeostasis and disease. NR4A1 exhibits pro-oncogenic activity in cancer cell lines derived from solid tumors and was overexpressed in tumors from lung, pancreatic, and colon cancer patients [29,30,31]. In lung cancer patients, high expression of NR4A1 was a prognostic indicator for decreased survival [15,23]. We investigated the function of NR4A1 in thyroid cancer cell lines using RNAi. We found that after knockdown of NR4A1, the rate of apoptosis significantly increased (Figure 3A–C). The regulation activity of the NR4A receptor is deemed to be ligand-independent, and the function of NR4A1 depends on the expression of the receptor and its posttranslational modification [18]. However, an increasing amount of evidence is showing that structurally diverse synthetic molecules directly interact with the ligand binding domain of NR4A1 and act as agonists or antagonists. A large number of small molecules have been identified, such as C-DIM, which is a NR4A1 antagonist, which induces apoptosis and activates growth inhibitory genes and pathways in breast cancer cells [32]. In order to search for NR4A1 small molecular ligands, 40 small molecule compounds were obtained from the Specs diversity library by virtual screening method. 

An antitumor test was completed for the small molecule compounds obtained from virtual screening. As a result, IMCA significantly inhibited the proliferation of thyroid cancer TT cell lines in a dose-dependent manner (Figure 2B). The antineoplastic activity of IMCA is similar to that observed after NR4A1 knockdown (Figure 2A). Several small molecules that significantly inhibit tumor cell proliferation and promote apoptosis have been reported in the literature, and these small molecules, including C-DIM, interact well with NR4A1 [33]. Using Autodock-1.5.6 software, the molecular docking of IMCA and NR4A1 was investigated. Consistent with the literature, the molecular docking results showed that IMCA combines well with NR4A1 (Figure 1).

**Figure 6:**
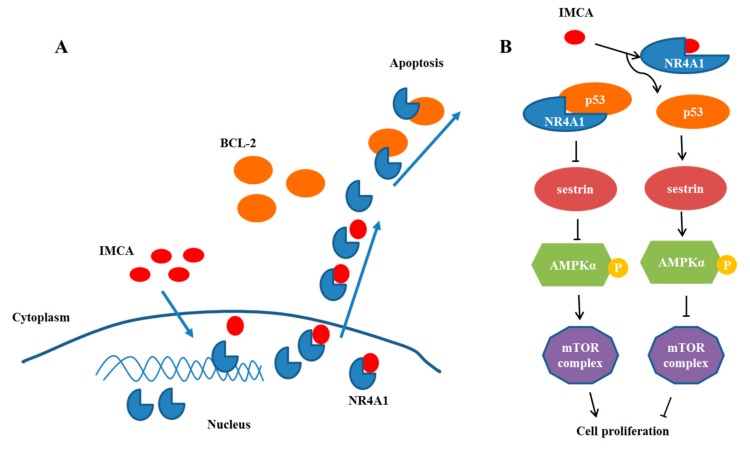
IMCA may be capable of inducing nuclear export of NR4A1 and inhibiting cell proliferation in thyroid carcinomas cell lines. (**A**) Illustration of the proposed impact of IMCA on apoptosis by inducing the nucleoplasm localization of NR4A1; (**B**) Illustration of the proposed impact of IMCA on cell proliferation through the interaction of NR4A1 and the NR4A1-p53-sestrins-AMPK-mTOR pathway in thyroid carcinomas cell lines.

To explore the effect of IMCA on thyroid cancer cell death, apoptosis was detected using Annexin V/PI double staining in TT cells. The apoptosis detection results showed that IMCA induces TT cell apoptosis in a dose-dependent manner (Figure 3D–J). These results are similar to those of NR4A1 knockdown (Figure 3A–C). In addition, the apoptosis-inducing effect of NR4A1 was related to its location in the cell. NR4A1 nuclear export was linked to the formation of a proapoptotic mitochondrial NR4A1-BCL-2 complex [31]. Cytosolic NR4A1 plays an important role in the mechanism of action of several pro-apoptotic antineoplastic agents including platinum-based drugs [34]. The effects of IMCA on the NR4A1 mitochondrial localization, were determined in the TT cell line, using immunofluorescence and Mito tracker red staining. As a result, IMCA was found to induce the NR4A1 nuclear export and mitochondrial location in a dose-dependent manner (Figure 4). These results demonstrate that the apoptosis regulated by IMCA may be related to the nuclear export and mitochondrial location of NR4A1in the medullary thyroid carcinoma cell lines (Figure 6A). 

The extranuclear activity of NR4A1 is a drug-induced response that invariably results in the induction of apoptosis; however, results of most knockdown or overexpression studies demonstrated the role of NR4A1 in cell proliferation, survival, migration and invasion. NR4A1 binds and inactivates p53 [35], whereas knockdown of NR4A1, or treatment of p53 wild-type (WT) lung cancer cells with an NR4A1 antagonist or transfection with siNR4A1, result in the activation of p53 and induction of sestrin1 and sestrin2 that activate AMPK and inhibit the mTOR pathway [15]. mTOR pathway inhibitors have been extensively developed for cancer chemotherapy [36,37]. The small molecular antagonists of NR4A1 represent a new class of mTOR inhibitor that block NR4A1-regulated mTOR activation in cancer cells that expressed p53 [15]. 

To determine the effect of compounds on the proliferation of thyroid cancer cells, we measured the expression and activation level of the genes related to the mTOR pathway in TT cells given intervention with different compounds. The results illustrated in Figure 5 show that IMCA inhibited the mTOR pathway in TT thyroid cancer cells. IMCA also induced sestrin1 and sestrin2 that activate AMPKa and inhibit mTOR, which is consistent with previous studies that NR4A1 antagonists also induced sestrin2, which activates AMPKa and inhibits mTOR pathway. These results demonstrate that the proliferation inhibited by IMCA may be related to the mTOR pathway (Figure 6B).

## 4. Materials and Methods

### 4.1. Virtual Screening

In this study, more than 200,000 small molecule compounds from the Specs compound library were screened for the spatial structure of NR4A1 (http://www.pdb.org; protein data bank, PDB ID 3V3Q) [24]. Initially, small molecular structures were optimized using the Ligprep module, including protonation, desalination, chiral isomers, and favorable conformations. A pharmacophore model between NR4A1 and small molecule compounds was established using the following five features: two receptor center oxygen atom-1-related Thr264, oxygen atom-2-related Glu114, a donor or acceptor center oxygen atom-3-related Arg184, two hydrophobic centers, and excluded volume. The optimized compounds were screened using this pharmacophore model and the screened compounds were docked with NR4A1. The highest value conformations docked with NR4A1 were screened again using the simplified pharmacophore model, which only retained the hydrogen bonds of the original pharmacophore model.

### 4.2. Computation-Based Molecular Modeling

The molecular modeling investigation was accomplished using Autodock version 1.5.6 (La Jolla, CA, USA). The crystal structure coordinates for the orphan nuclear receptor NR4A1 [24] were downloaded from the Protein Data Bank (PDB ID 3V3Q). To perform subsequent docking experiments, water and small molecule ligands were removed from the downloaded crystal structure of NR4A1. The semi-flexible docking algorithm was used to predict the binding orientation of IMCA with the ligand binding site of NR4A1. The docking between the different conformations of IMCA and the ligand binding site of NR4A1 was calculated 25,000,000 times.

### 4.3. Chemicals and Reagents

IMCA was designed and synthesized by Tao Su Biochemical Technology Co. Ltd. (Shanghai, China). AMPK, p-AMPK, and p-P70S6K antibodies were purchased from Cell Signaling Technology (Boston, MA, USA). BCL-2, BAX, P70S6K, β-actin, and Alexa Fluor 488-conjugated affinipure goat anti-rabbit IgG were purchased from Proteintech Co. Ltd. (Wuhan, China). The DAPI Staining Kit, annexin V/PI double staining apoptosis detection kit, and MTT cell proliferation and cytotoxicity detection kit were purchased from Keygen Biotech Co. Ltd. (Nanjing, China). Mito Tracker^TM^ Red CMXRos-Special Pcakaging was purchased from Invitrogen (Carlsbad, CA, USA). PCR primers were designed and synthesized by Sangon Biothec Co. Ltd. (Shanghai, China). The SYBR green PCR Master Mix was purchased from Thermo Scientific (Waltham, MA, USA).

### 4.4. Cell Lines and Cell Culture

The TT cell line was purchased from a typical cell culture collection committee of the Chinese academy of sciences library (Shanghai, China). The cells were maintained in Roswell Park Memorial Institute 1640 (RPMI 1640) medium containing 0.22% sodium bicarbonate, 0.011% sodium pyruvate, 10% fetal bovine serum (FBS), 100 U/mL penicillin, and 100 μg/mL streptomycin. Cells were cultured at 37 °C in a humidified atmosphere of 5% carbon dioxide. The solvent dimethyl sulfoxide (DMSO) used in the experiments was less than 0.1%.

### 4.5. siRNA Assay

Short-interfering RNA (siRNA) specific for NR4A1 was obtained from GenePharma Company (Shanghai, China). TT thyroid cancer cells were seeded in six-well plates in RPMI 1640 medium supplemented with 10% FBS. As previously described [38], siRNAs were transfected in TT thyroid cancer cells using Lipofectamine 3000 (Invitrogen) reagent according to the manufacturer’s protocol. Cells were collected for Western blot analysis 48 h after transfection. The siNR4A1 sequence was CAGUGGCUCUGACUACUAUTTAUAGUAGUCAGAGCCACUGTT.

### 4.6. MTT Assay

Exponentially growing cells were seeded into 96-well plates with a density of 5 × 10^3^ cells per mL, incubated for 12 h at 37 °C, and subsequently treated with siNR4A1 or different concentrations of IMCA for 48 h. A total of 10 µL MTT solution (10 mg/mL in PBS) was added to the 96-well plates after the incubation. After 4 h of incubation at 37 °C, the preformed MTT-formzan crystals were dissolved using 100 µL DMSO, and the absorbance of the samples was measured using a controlled microplate reader (Labexim LMR-1) at the length of 570nm wave. The resultant MTT data were expressed as the percentage of the untreated control sample and subsequently fitted to a sigmoidal dose-response curve.

### 4.7. Annexin V and PI Staining

According to the kit instruction, cell apoptosis was evaluated by flow cytometry using an Annexin V-FITC/PI apoptosis detection kit [39]. Briefly, TT cells were seeded in six-well plates at a density of 1 × 10^4^ cells per well for 12 h and were then treated with siNR4A1 or different concentrations of IMCA for another 48 h. Untreated cells served as the negative controls. Afterward, the cells were digested by trypsin without ethylenediaminetetraacetic acid (EDTA), collected, and washed three times with PBS diluted in 1× Annexin binding buffer (500 μL). For each sample, 5 μL (2.5 μg/mL) of Annexin-V-FITC and 5 μL (50 μg/mL) propidium iodide were added to the cell suspension and incubated for 15 min at room temperature (25 °C) in the dark. Cell death by apoptosis was scored by quantifying the population of Annexin V-FITC-positive cells for 10,000 events. Flow cytometry data were plotted and analyzed by the fluorescence activated cell-sorting (FACS-Vantage) system using Cell Ouest software (Becton-Dickinson, San Jose, CA, USA) within one hour after staining.

### 4.8. Immunofluorescence Assay

As previously described [40], for each experimental group, 5 × 10^3^ TT cells were cultured on coverslips placed in 24-well plates. Cells at 30% confluency were exposed to different concentrations of IMCA (50 μM, 25 μM, 12.5 Μm, and 6.25 μM) for 48 h. The storage solution of Mito Tracker^TM^ Red CMXRos-Special Pcakaging is diluted to 200 nM, and the 200 nM working solution was used to incubate TT cells for 30 min. After incubation, cells were washed with 1 ×PBS (138 mM NaOH, 3 mM HCl, 8.1 mM Na_2_HPO_4_, and 1.5 mM KH_2_PO_4_; pH 7.4) and were fixed with neutral formalin (4% formaldehyde in PBS) for 15 min, followed by three washes with 1 × PBS. Then, TT cells were permeated with 0.2% Triton X-100 for 5 min at room temperature. Subsequently, cells were blocked for 15 min with universal blocking reagent and incubated overnight with the respective primary antibody. Following three washes for 5 min with PBS-T (0.1% Tween-20), cells were incubated with goat anti-rabbit IgG conjugated Alexa Fluor 488 for one hour and 4’,6-diamidino-2-phenylindole (DAPI) for 10 min at room temperature. Photomicrographs were obtained under a fluorescence optical microscope (Nikon, Tokyo, Japan). 

### 4.9. Real-Time PCR 

Total RNA was extracted using Trizol reagent. Reverse transcription was performed in a 20 μL reaction mixture. PCR reactions were run on an ABI 7500 Fast Real-Time PCR System (Applied Biosystems, Waltham, MA, USA). The primer sequences used in this study were as follows: sestrin1: 5′-AGTGGGGAGTGAAGACGC-3′ (forward) and 5′-GGCCCATCCATTTGCAGTAGA-3′ (reverse); sestrin2: 5′-AAGGACTACCTGCGGTTCG-3′ (forward) and 5′-GCCCAGAGGACATCAGTGC-3′ (reverse); and β*-actin*: 5′-CATGTACGTTGCTATCCAGGC-3′ (forward) and 5′-CTCCTTAATGTCACGCACGAT-3′ (reverse). All samples were assayed in triplicate.

### 4.10. Western Blotting

Cells were harvested and lysed in a high salt lysis buffer with a protease inhibitor cocktail. Samples were quantitated using a Bradford reagent. Lysates were separated by a denatured sodium dodecyl sulfate polyacrylamide gel electrophoresis (SDS-PAGE) and transferred onto a polyvinylidene fluoride (PVDF) membrane through wet electroblotting. The membrane was incubated overnight at 4 °C with the primary antibodies. Following incubation with a secondary antibody at room temperature for 2 h, Western blot analysis was performed as described and detected using a chemiluminescence analyzer (Amerisham Biosciences, Boston, MA, USA).

### 4.11. Statistical Analysis

Statistical significance of differences between the groups was determined using the t-test. The figures were drawn with GraphPad Prism 6 (GraphPad Software, La Jolla, CA, USA). The data are expressed as means ± standard deviation. A *p* value of less than 0.05 was considered statistically significant.

## 5. Conclusions

In summary, we found a new antineoplastic compound, IMCA, can induce tumor cell death in a dose-dependent manner. IMCA may be a specific antagonist of NR4A1 through the nuclear export of NR4A1 and the p53-sestrins-AMPK-mTOR signaling pathway, meaning it may be a promising drug candidate to treat thyroid carcinomas.

## Supplementary Materials

The supplementary materials are available online, Figure S1: Compared with the siCtl groups, the expression of NR4A1 was significantly reduced in the siRNA processing groups.
