# Supplementary material for: New Drug Candidate Targeting the 4A1 Orphan Nuclear Receptor for Medullary Thyroid Cancer Therapy
Source: Molecules. 2018 Mar 2;23(3):565. doi: 10.3390/molecules23030565 (PMC6017334; doi:10.3390/molecules23030565)
Supplement: Supplementary file 1 [file molecules-23-00565-s001.pdf]

We identified the effect of IMCA on cell proliferation in ARO cell lines, which is an anaplastic thyroid cancer cell line (Figure 1).

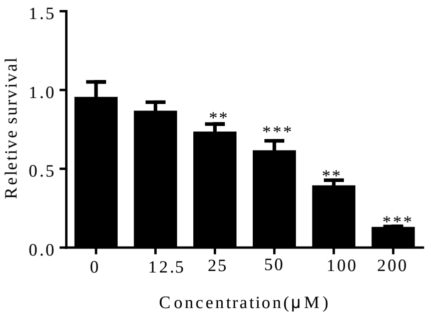

Figure 1. IMCA inhibits cell growth in ARO cell lines.

The immunofluorescence and mitochondrial staining experiments showed that NR4A1 was located on the mitochondria (Figure 2).

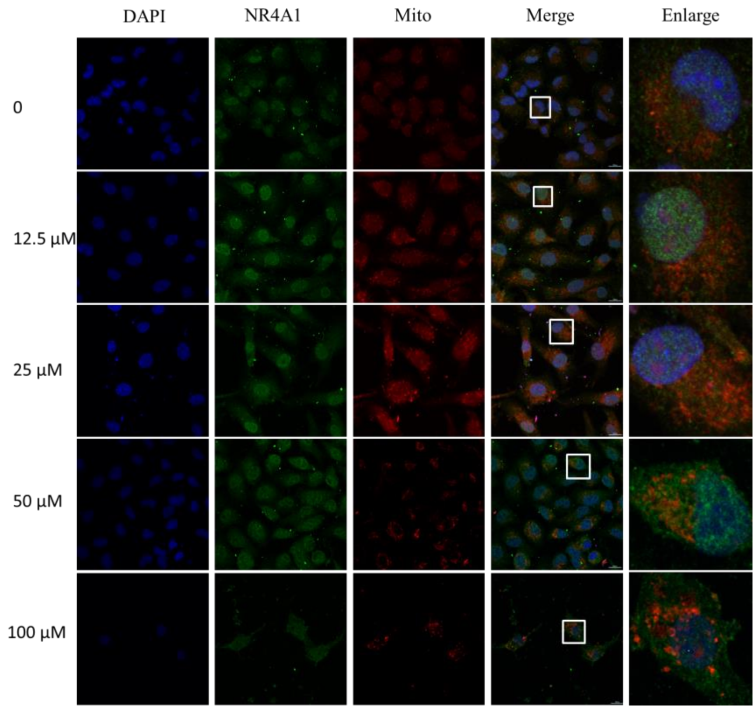

Figure 2. Immunofluorescence and mitochondrial staining assay for the localization of NR4A1 into mitochondria induced by IMCA in TT cells. TT cells treated with different concentration of IMCA for 48 hours, are fixed with neutral formalin and incubated with NR4A1 antibody. MitoTracker™ Red and DAPI are added. Fluorescence microscopy shows that the nucleus dyed with DAPI is shown as blue fluorescence, NR4A1 immunofluorescence is shown as green and mitochondrion is shown as red. Merged images shows that the NR4A1 is induced the localization into mitochondria by IMCA. The graphs in the last column are the magnified images of the white line frame in the fourth column.
